# Induced hepatic stem cells maintain self-renewal through the high expression of Myc coregulated by TET1 and CTCF

**DOI:** 10.1186/s13578-022-00883-7

**Published:** 2022-09-02

**Authors:** Chen Wang, Xinlu Yu, Sai Ding, Yang Liu, Hongxia Zhang, Jingbo Fu, Bing Yu, Haiying Zhu

**Affiliations:** 1 Department of Cell Biology, Naval Medical University (Second Military Medical University), 800 Xiangyin Road, Shanghai, 200433 People’s Republic of China; 2 Department of Laboratory Medicine, PLA 960th Hospital, Jinan, 250031 China

**Keywords:** Tet1, Ctcf, Myc, Induced hepatic stem cell, Self-renewal

## Abstract

**Background:**

Induced hepatic stem cells (iHepSCs) with the capacities of self-renewal and bidifferentiation into hepatocytes and cholangiocytes were generated from mouse embryonic fibroblasts (MEFs) by lineage reprogramming in our previous research. However, the mechanism of iHepSC self-renewal has not been elucidated. Active demethylation regulated by Tet1 plays an important role in the self-renewal of stem cells, including pluripotent stem cells and adult stem cells. Here, we investigated the role and mechanism of Tet1-regulated demethylation in the self-renewal of iHepSCs.

**Methods:**

The methylation levels and the expression of Tet1 in iHepSCs and MEFs were analyzed by immunofluorescent staining, quantitative reverse transcription PCR and western blotting. Then, the effects of Tet1 knockdown on the proliferation and self-renewal of iHepSCs were analyzed by CCK8, colony formation, and sphere formation assays. The mechanism by which Tet1 regulates the self-renewal of iHepSCs was investigated by chromatin immunoprecipitation, bisulfite sequence PCR, and methylation-sensitive restriction endonuclease-PCR.

**Results:**

The high level of 5hmC and the low level of 5mC in iHepSCs were accompanied by high expression of Tet1. After Tet1 expression was knocked down by shRNA in iHepSCs, the proliferation and self-renewal capacities were inhibited, and the expression of Myc was also decreased. The higher expression level of Myc in iHepSCs maintained its self-renewal and was regulated by Tet1, which directly binds to CBS-1 and site A regions of the Myc promoter and demethylates the CpG cytosine. In addition, CTCF also binds to the CBS-1 and site A regions of the Myc promoter and regulates Myc expression along with TET1.

**Conclusion:**

The self-renewal of iHepSCs was maintained by the higher expression of Myc, which was coregulated by TET1 and CTCF. This study may provide new insights into the self-renewal of stem cells, which can promote the research and application of ‘reprogrammed’ stem cells.

**Supplementary Information:**

The online version contains supplementary material available at 10.1186/s13578-022-00883-7.

## Introduction

Many different types of tissue-specific progenitor/stem cells were generated from fibroblasts by direct lineage reprogramming [1]. In our previous study, we established induced hepatic stem cells (iHepSCs) directly converted from MEFs through overexpression of two liver organogenesis transcription factors, Hnf1b and Foxa3 [2]. Further studies indicated that iHepSCs could be stably expanded (exceeding 50 passages) in vitro and maintained their normal chromosomal numbers and the capacity of bipotential differentiation into either hepatocytes or cholangiocytes without obvious senescence [3]. Obviously, iHepSCs maintained the capacity of self-renewal, which was defined as division with maintenance of an undifferentiated state, during long-term culture in vitro. However, the exact mechanism through which iHepSCs maintain the capacity of self-renewal has not been elucidated.

Self-renewal of stem cells not only requires cell cycle control but also maintains multipotency or pluripotency depending on the stem cells, in which epigenetic regulatory mechanisms, such as DNA methylation, RNA processing and histone modification, are involved [4]. DNA methylation affects gene expression through the conversion between methylation and demethylation, which usually occurs in the context of CpG dinucleotides [5]. DNA demethylation can occur either passively or actively. The passive process takes place during DNA replication, while active DNA demethylation requires the action of enzymes in the ten-eleven translocation (TET) protein family, including TET1, TET2, and TET3 [6]. TET enzymes, 2-oxoglutarate, oxygen- and iron-dependent dioxygenases, are able to catalyze the oxidation of 5-methylcytosine (5-mC) into 5-hydroxymethylcytosine (5-hmC). This reaction is the initial step in a series of events that ultimately lead to DNA demethylation [7–9]. In addition to depending on the oxidation activity, TET proteins also bind to the promoter region of target genes to regulate their expression as transcription factors or complexes formed with other transcription factors [10, 11].

Recently, the functions of TET family proteins have gained more attention in epigenetic reprogramming during development and in stem cell research [12–15]. Among the TET protein family, TET1 is not only well known to be highly expressed in embryonic stem cells and neurons but also essential for the establishment of induced pluripotent stem cells [16, 17]. Furthermore, TET1 has been reported to be involved in the maintenance of self-renewal in stem cells, including embryonic stem cells and tissue specific stem cells [13, 18–21]. In this study, we compared the level of methylation and the expression level of Tet1 in iHepSCs and MEFs. Then, the functions of Tet1 in the maintenance of self-renewal in iHepSCs were explored by downregulation with shRNA, and the mechanism of self-renewal of iHepSCs was also investigated. This study may provide new insights into the self-renewal of stem cells, which can promote the research and application of stem cells.

## Materials and methods

### Cell culture and cell differentiation

MEFs and 293T cells were cultured in DMEM supplemented with 10% FBS, 100 U/mL penicillin and 100 µg/mL streptomycin. iHepSCs were cultured in SCM-A medium as previously reported [2]. Hepatic differentiation and cholangiocytic differentiation of iHepSCs were induced as described in our previously reported procedures [2, 3]. TET-IN-C35 (AOB11121) was purchased from AOBIOUS, Inc. and was dissolved in DMSO to prepare a 5 mM stock solution. For the low-density lipoprotein (LDL) uptake assay, hepatic differentiated cells were incubated with 10 µg/mL DiI AcLDL (Invitrogen) for 4 h at 37 °C and then observed by fluorescence microscopy. Glycogen storage was detected by a periodic acid Schiff (PAS) staining kit (SJ1269, ShuangJian Biotech, Shanghai) according to the manufacturer’s user manual.

### Lentivirus production and infection

The open reading frame (ORF) of Myc was separately cloned into the pLVX-IRES-ZsGreen1 (Clontech) lentivirus vector. Short hairpin RNAs (shRNAs) targeting Tet1, Myc and Ctcf were separately cloned into the pLKO.1 lentiviral vector (#10878, Addgene), which can induce stable and long-term gene silencing in mammalian cells [22]. The target sequences for the shRNAs are shown in Additional file 1: Table S1. Lentivirus was packaged in 293T cells that were cotransfected with lentivirus expression vector and lentiviral packaging vectors, including psPAX2 (#12260, Addgene) and pMD2. G (#12259, Addgene). Viral supernatant was harvested at 48 and 72 h post-transfection. After the lentiviral particles were concentrated by ultracentrifugation at 45,000×*g* for 2 h, the pellets were resuspended in PBS, and the titer was determined with a TransLv™ Lentivirus qPCR Titration Kit (TransGen Biotech, China). Cells were infected with virus at a multiplicity of infection (MOI) of 10.

### Quantitative reverse transcription PCR (qRT-PCR)

Total RNA was extracted using TRIzol reagent (TaKaRa), and the first cDNAs were synthesized using MMLV Reverse Transcriptase (Promega) according to the manufacturer’s guidelines. qRT-PCR analysis was performed using LighterCylcer® 96 (Roche) with SYBR Premix Ex Taq reagent (TaKaRa). All samples were examined in triplicate. GAPDH was used as an internal control. The primers are listed in Additional file 1: Table S2.

### Cell proliferation assay

Cell proliferation assays were carried out as previously described [3]. Briefly, 1 × 10^3^ cells per well were plated on 96-well plates and incubated for 24 h. Then, 10 µL CCK8 reagent (Dojindo, Japan) was added to each well and incubated for 4 h at 37 °C. The optical density value was measured at 450 nm every day for 5 days.

### Cell sphere formation assay

Adherent cells were digested and counted, and 1 × 10^4^ cells were seeded into an Ultra-Low Attachment 6-well plate (#3471, Corning) per well. The cells were cultured at 37 °C for 7 days to form the cell spheres. The number of cell spheres was counted under a microscope.

### Colony formation assay

iHepSCs were seeded at a density of 200 cells per well into 6-well plates and incubated for 7 days in a cell incubator at 37 °C. Then, the cells were fixed with 4% PFA and stained with crystal violet staining solution (#Y1232, Yuxiu Biotech, China). The number of colonies containing more than 50 cells was counted.

### Tet enzyme activity assay

Nuclear proteins from iHepSCs and MEFs were prepared with a Nuclear Extraction Kit (ab113474, Abcam) and quantitated with Optiblot Bradford Reagent (ab119216, Abcam). The activity of the Tet family of 5mC hydroxylases, including TET1, TET2 and TET3, was detected by a TET Hydroxylase Activity Quantification kit (Colorimetric) (ab156912, Abcam) according to the manufacturer’s instructions. The amount of nuclear extracts for each assay was 10 µg per sample. The optical value was measured at 450 nm with an optional reference wavelength of 655 nm by Molecular Devices Spectra MAX190. The activity of the Tet enzyme was calculated as follows: Tet activity (OD/min/mg) = (sample OD – blank OD)/(protein amount (µg) × 90(min)) × 1000.

### Chromatin immunoprecipitation

Chromatin immunoprecipitation (ChIP) assays were performed using an EZ-ChIP kit (Millipore, USA) according to the manufacturer’s protocol. Briefly, cells were first fixed with 1% formaldehyde to generate DNA‒protein crosslinks. Then, the cells were lysed and sonicated to generate chromatin fragments of 200 to 300 bp, and the cell lysates were immunoprecipitated with the target protein antibody. After immunoprecipitation, genomic DNA was isolated, and bound protein was digested with proteinase K. qRT-PCR was performed using the primers listed in Additional file 1: Table S3.

### Immunocytofluorescence staining

Immunocytofluorescence staining was performed as reported previously [2]. In brief, the fixed cells were permeabilized with 0.3% Triton X-100 for 5 min and then blocked with 1% BSA for 1 h. Cells were incubated with primary antibody overnight at 4 °C and then with secondary antibody. Nuclei were counterstained with DAPI. For negative controls, primary antibodies were omitted, and only secondary antibodies were used.

### Western blot and dot blot assays

Western blotting assays were performed as previously described [23]. A dot blot assay was performed as follows: the sample DNA was diluted to final DNA concentrations of 1000 ng/µL, 500 ng/µL and 200 ng/µL. Two microliters of diluted DNA was dropped onto a cellulose acetate membrane, which was immersed in 6× SSC for 5 min. Then, the membrane, dried in an oven at 70 °C, was blocked with 5% skimmed milk in TBS-T for 2–4 h. After probing with 5hmC or 5mC antibody, the membrane was incubated with HRP-conjugated goat anti-mouse IgG, and illuminant (#T7103Q, Takara) was used to expose the spot marks. The primary antibodies used in immunocytofluorescence staining, dot blot and western blot are listed in Additional file 1: Table S4.

### Bisulfite sequence PCR (BSP)

The genomic DNA was extracted from cells with a QIAamp DNA Mini Kit (51304, QIAGEN), and bisulfite-mediated conversion of cytosine to uracil was performed using a MethylEasy Xceed Rapid DNA Bisulphite Modification Kit (ME002, Human Genetic Signatures). Amplification PCR was performed in a 50 µL reaction volume containing 100 ng genomic DNA template, 0.4 µM of each primer, 0.3 mM dNTP and 1.25 U TaKaRa EpiTaq™ HS (R110Q, TaKaRa) on a ProFlex PCR system with the following program: 40 cycles of denaturing for 10 s at 98 °C, annealing for 30 s at 55 °C and extension for 30 s at 72 °C. The primers were designed with Methprimer on the website of http://www.urogene.org/methprimer/index.html. The sequences of the primers are listed in Additional file 1: Table S5. The PCR products were purified with a MinElute PCR Purification Kit (28004, QIAGEN). The purified fragments were cloned into the pMD™18-T vector (6011, TaKaRa). Ten clones from three independent amplification experiments were picked and sequenced. The sequence results were processed by online QUMA software [24].

### Methylation-sensitive restriction endonuclease-PCR (MSRE-PCR)

The genomic DNA extracted from cells was digested with AciI (R0551 V, New England BioLabs) and HpaII (R0171 V, New England BioLab), respectively. Genomic DNA digested with EcoRI was used as the control. The purified digested genomic DNA with a MinElute Gel Extraction Kit (28604, QIAGEN) was used as a template for PCR amplification. The sequences of the primers for MSRE-PCR are listed in Additional file 1: Table S6. The PCR products were visualized by electrophoresis in a 2% agarose gel.

### Statistical analysis

All statistical analyses were performed using the GraphPad Prism (version 7.0) program. The data are shown as the mean and standard error of the mean. Statistical methods are indicated in the figure legends, and differences were considered statistically significant when P < 0.05.

## Results

### The levels of 5hmC and the expression of Tet1 in iHepSCs are higher than those in MEFs

**Fig. 1:**
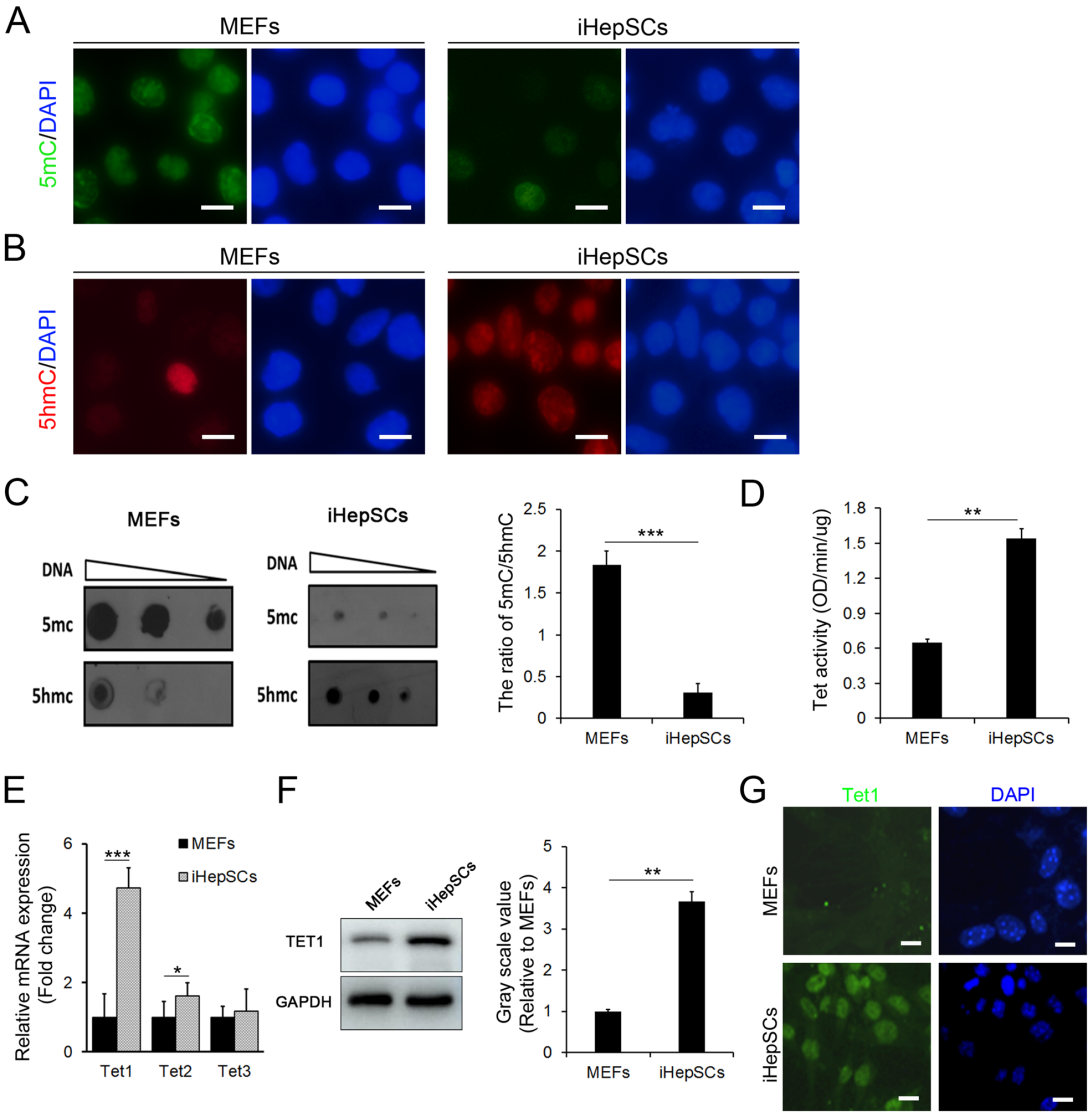
iHepSCs exhibit higher levels of 5hmC and Tet1 expression. **A**, **B** Immunocytofluorescence staining showed that the level of 5mC was much lower in iHepSCs than in MEFs (**A**), while the level of 5hmC was much higher in iHepSCs than in MEFs (**B**). **C** Dot blot assay showing the levels of genomic 5mC and 5hmC in MEFs and iHepSCs. **D** Tet enzyme activity increased dramatically in iHepSCs compared with MEFs. **E** The results of qRT-PCR showed that the expression level of Tet1 mRNA in iHepSCs was significantly higher than that in MEFs. **F**, **G** Western blot (**F**) and immunocytofluorescence staining (**G**) assays showed that Tet1 expression in iHepSCs was significantly higher than that in MEFs. The data are shown as the mean ± SEM, n = 3, Student’s t test, *P < 0.05, **P < 0.01, ***P < 0.001

In previous studies, we proved that iHepSCs maintained the capacity of self-renewal after extensive expansion [3]. While DNA methylation was reported to be involved in the maintenance of self-renewal and stemness of stem cells [25], the levels of DNA methylation (5mC) and hydroxymethylation (5hmC) in iHepSCs and the ancestor cells, MEFs, were examined. The results of immunocyte fluorescent staining (Fig. 1A, B) and dot blot assays (Fig. 1C) showed that the level of 5mC was much lower while 5hmC was much higher in iHepSCs than in MEFs. Because active DNA demethylation is often mainly mediated by the TET enzymes that modify the methyl group by oxidizing 5 mC to 5hmC [26], the enzyme activity of TET in iHepSCs was measured. The results showed that the enzyme activity of TET in iHepSCs was significantly higher than that in MEFs (Fig. 1D). Consistent with this finding, the expression of Tet1 and Tet2 in iHepSCs was significantly higher than that in MEFs. Specifically, the expression of Tet1 in iHepSCs was obviously higher than the expression of Tet2 and Tet3 (Fig. 1E). We further used western blot and immunocyte fluorescent staining to demonstrate that the expression of TET1 in iHepSCs was significantly higher than that in MEFs (Fig. 1F, G).

### Tet1 maintains a high level of 5hmC in iHepSCs

**Fig. 2:**
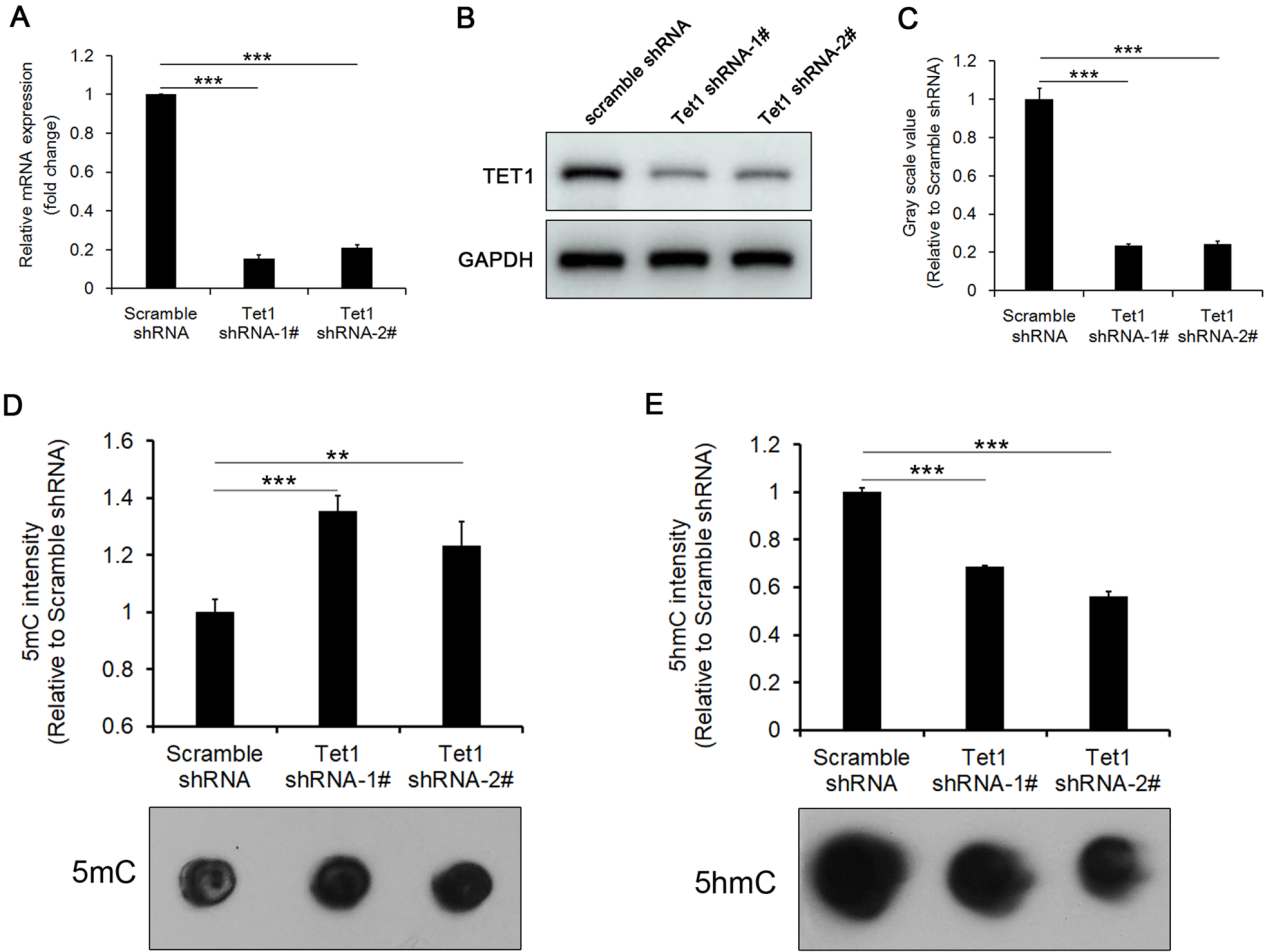
Tet1 maintains a high level of 5hmC in iHepSCs. **A** The results of qRT-PCR showed that the expression of Tet1 mRNA was repressed by shRNA. **B** The expression of Tet1 protein was determined by Western blot. **C** The quantification of the relative intensities of blots (**B**) showed that the protein expression of Tet1 was significantly downregulated. **D** The results of dot blot showed that the level of genomic 5mC was upregulated when the expression of Tet1 was downregulated. Each sample was loaded with 500 ng genomic DNA. **E** The results of dot blot showed that the level of genomic 5hmC was downregulated when the expression of Tet1 was inhibited. Each sample was loaded with 500 ng genomic DNA. The data are shown as the mean ± SEM, n = 3, Student’s t test, **P < 0.01, ***P < 0.001

To illustrate that Tet1 is involved in maintaining a high level of 5hmC in iHepSCs, first, the expression of Tet1 in iHepSCs was knocked down by shRNA (Fig. 2A–C). After the inhibition of Tet1 expression in iHepSCs, the level of genomic 5mC was upregulated (Fig. 2D), while the level of genomic 5hmC was downregulated (Fig. 2E). Furthermore, we found that there were no significant differences in the expression levels of Tet1 between the third generation (P3) and tenth generation (P10) after virus infection (Additional file 1: Fig. S1A–C), indicating that lentivirus-mediated shRNA can maintain the low expression of Tet1 in iHepSCs for a long time. Meanwhile, the results of dot blot also showed that there was no significant difference in the global level of genomic 5hmC between the P3 and p10 Tet1-KD iHepSCs (Additional file 1: Fig. S1D, E).

### Tet1 maintains the capacity of self-renewal of iHepSCs

**Fig. 3:**
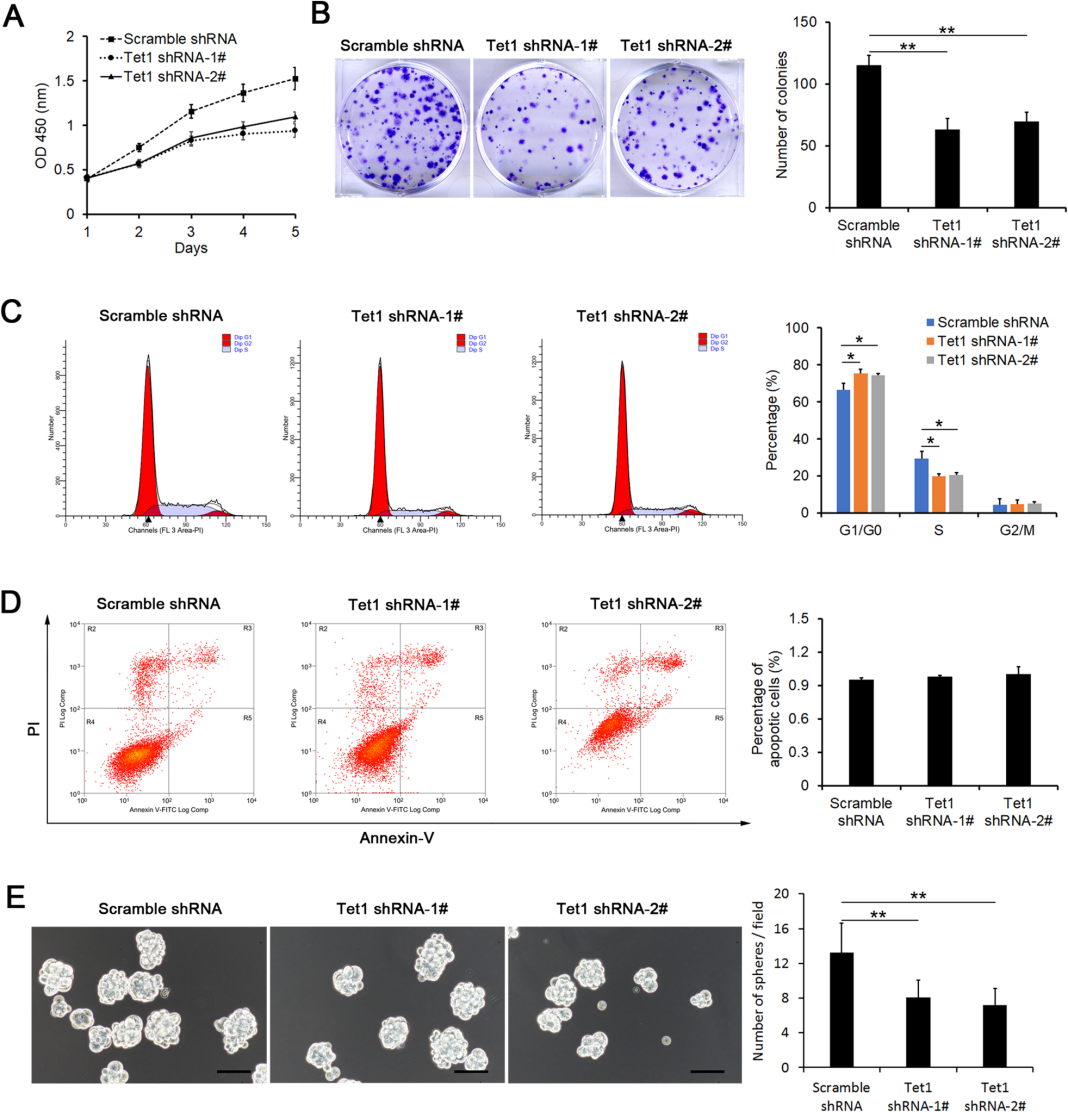
Tet1 maintains the self-renewal of iHepSCs. **A** CCK 8 assay showed that the proliferation capacity of iHepSCs was inhibited after downregulating the expression of Tet1 with shRNA. **B** Representative images of colony formation of iHepSCs-scramble and Tet1-KD iHepSCs (left), and the numbers of colonies were significantly reduced in iHepSCs after downregulating the expression of Tet1 (right). **C** Representative plots (left) and statistical chart (right) of the percentage of G_0_/G_1_, S, and G_2_M cells of iHepSCs-scramble and Tet1-KD iHepSCs. The cell cycle of Tet1-KD iHepSCs exhibited obviously arrested G_0_/G_1_ compared to iHepSCs-scramble. **D** Representative plots (left) and statistical chart (right) of apoptosis of iHepSCs-scramble and Tet1-KD iHepSCs. **E** Representative images of spheres of iHepSCs-scramble and Tet1-KD iHepSCs (left), and the numbers of spheres were significantly reduced in iHepSCs after downregulating the expression of Tet1 (right). The data are shown as the mean ± SEM, n = 3, Student’s t test, *P < 0.05, **P < 0.01

We further detected the impacts of Tet1 on the characterization of iHepSCs. After the expression of Tet1 was downregulated by shRNA in iHepSCs, the capacities of cell proliferation and colony formation were significantly reduced (Fig. 3A, B). The cell cycle and apoptosis assays showed that inhibition of Tet1 expression promoted G1/G0 phase arrest in iHepSCs (Fig. 3C), whereas the percentage of apoptotic cells did not change obviously (Fig. 3D). In addition, sphere-forming assays have been widely used to evaluate the self-renewal and differentiation of stem cells at the single-cell level in vitro [27]. Here, the results of the cell sphere formation assay showed that the rate of sphere formation in Tet1-downregulated iHepSCs was obviously lower than that in control iHepSCs (Fig. 3E). Meanwhile, the downregulation of Tet1 expression in iHepSCs did not affect the differentiation potential into hepatocytes (Additional file 1: Fig. S2) and cholangiocytes (Additional file 1: Fig. S3). Together, these results indicated that the inhibition of Tet1 expression suppressed the capacity of self-renewal of iHepSCs.

### Myc contributes to the self-renewal of iHepSCs

**Fig. 4:**
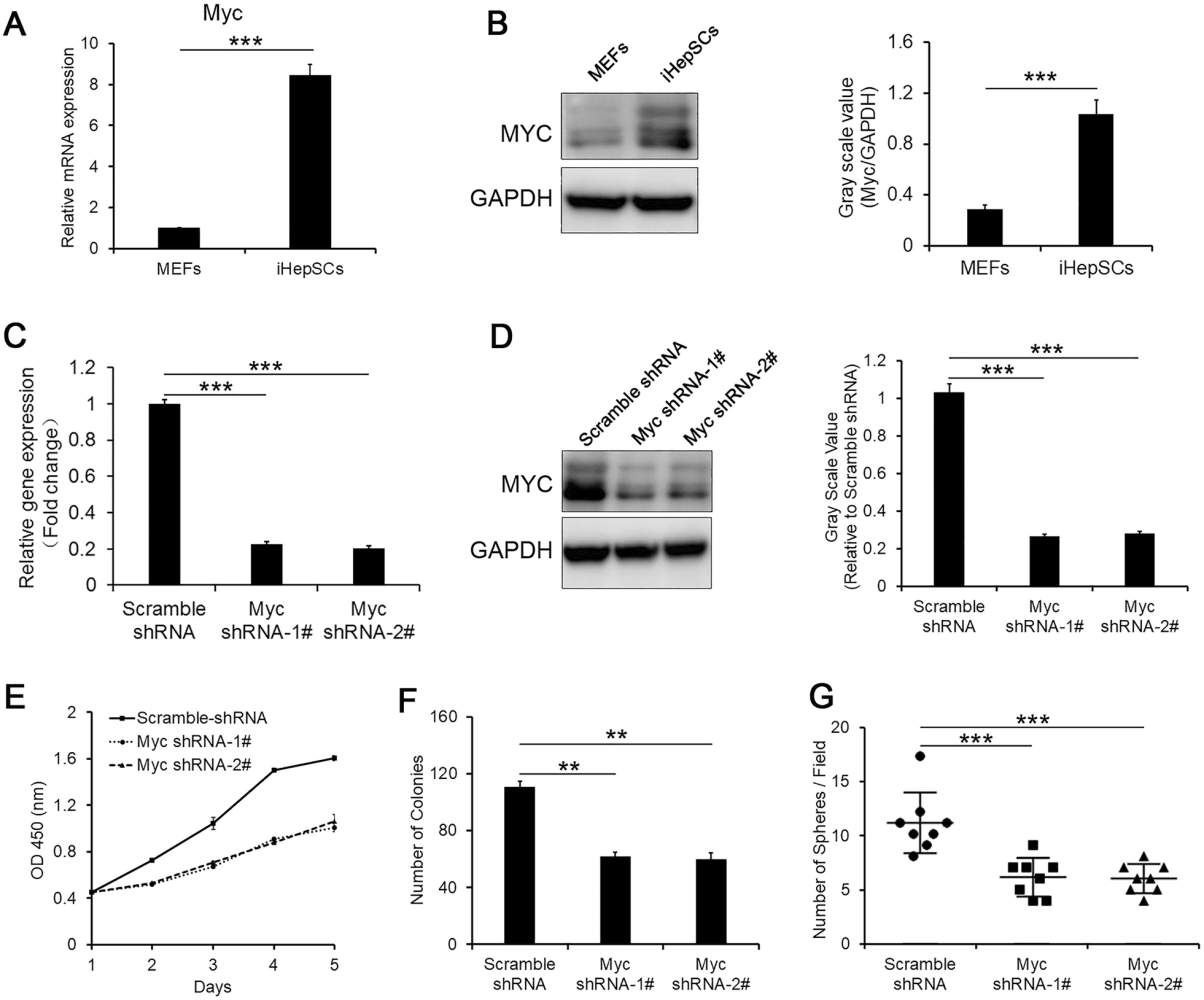
Myc maintains the self-renewal of iHepSCs. **A**, **B** The expression of Myc in iHepSCs was higher than that in MEFs, as detected by qRT-PCR (**A**) and Western blotting (**B**). **C**, **D** The expression of Myc in iHepSCs and Myc knockdown iHepSCs was detected by qRT-PCR (**C**) and Western blotting (**D**). **E** The proliferation capacity of iHepSCs was inhibited after the expression of Myc was knocked down by shRNA. **F** The colony number of iHepSCs was significantly reduced after the expression of Myc was knocked down by shRNA. **G** The sphere numbers of iHepSCs were reduced after the expression of Myc was knocked down by shRNA. The data are shown as the mean ± SEM, n = 3, Student’s t test, **P < 0.01, ***P < 0.001

Considering that the inhibition of Tet1 expression promotes G0/G1 phase arrest, Myc protein is well known to promote cell division and accelerate cell entry to S phase from G0/G1 phase [28]. We speculated that Myc may be involved in the self-renewal of iHepSCs. First, we compared the expression levels of Myc between iHepSCs and MEFs by qRT-PCR and western blotting, and the results indicated that the expression level of Myc in iHepSCs was obviously higher than that in MEFs (Fig. 4A, B). To further elucidate whether Myc is involved in the self-renewal of iHepSCs, we established Myc knockdown iHepSCs by shRNA (Fig. 4C, D) and observed that proliferation (Fig. 4E), colony formation (Fig. 4F) and cell sphere formation (Fig. 4G) were all significantly decreased compared with those in control iHepSCs due to the inhibition of Myc expression.

## Tet1 maintains self-renewal by targeting Myc in iHepSCs

**Fig. 5:**
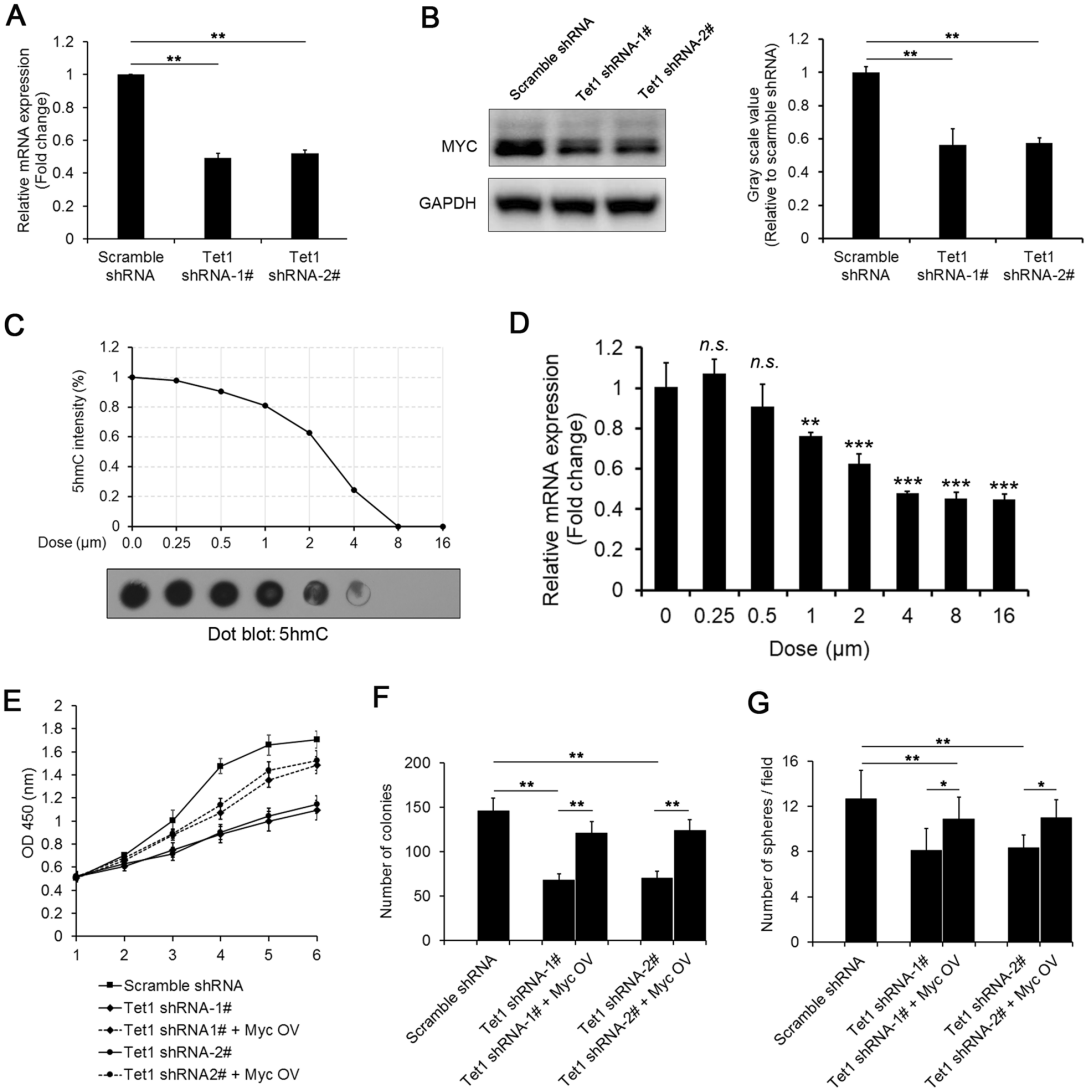
Tet1 maintains self-renewal by targeting Myc in iHepSCs. **A**, **B** The expression of Myc was reduced after the expression of Tet1 was knocked down by shRNA, as detected by qRT-PCR (**A**) and Western blotting (**B**). **C** The level of 5hmC in iHepSCs treated with the indicated dosage of TET-IN-C35 for 6 days was analyzed by dot blot. Each sample was loaded with 500 ng genomic DNA. **D** The expression level of Myc in iHepSCs treated with TET-IN-C35 was measured by qRT-PCR analyses. **E** The overexpression of Myc recovered the proliferation capacity of Tet1-KD iHepSCs. **F** The overexpression of Myc increased the colony numbers of Tet1-KD iHepSCs. **G** The overexpression of Myc increased the sphere numbers of Tet1-KD iHepSCs. The data are shown as the mean ± SEM, n = 3, Student’s t test (**A**, **B**, **F** and **G**) and Dunnett’s test (**D**), *P < 0.05, **P < 0.01, ***P < 0.001, *n.s.*, not significant

To reveal the possible connection between Tet1 and Myc in maintaining the self-renewal of iHepSCs, we investigated the expression level of Myc after Tet1 was knocked down. The results of qRT-PCR and western blotting showed that the expression of Myc was significantly reduced with the inhibition of Tet1 expression by shRNA (Fig. 5A, B). Next, we used TET-IN-C35, a first-in-class TET inhibitor that specifically blocks TET enzyme catalytic activities [29], to treat iHepSCs, and the results showed that the level of genomic 5hmC decreased in a dose-dependent manner (Fig. 5C). Correspondingly, the expression of Myc also decreased. However, when the concentration of TET-IN-C35 exceeded 4 µM, the expression of Myc no longer decreased with increasing drug dose (Fig. 5D). The results above suggested that Tet1 may target Myc expression to maintain the self-renewal of iHepSCs. Moreover, as important supportive data for the hypothesis, the overexpression of Myc by lentivirus rescued the downregulation of Myc in Tet1 knockdown iHepSCs and further recovered proliferation (Fig. 5E), colony formation (Fig. 5F) and cell sphere formation (Fig. 5G). Overall, these results suggested that Tet1 could maintain the self-renewal of iHepSCs by targeting Myc.

### Tet1 regulates the expression of Myc by directly binding to the CBS-1 and site A regions of the Myc promoter and demethylating the CpG cytosine

**Fig. 6:**
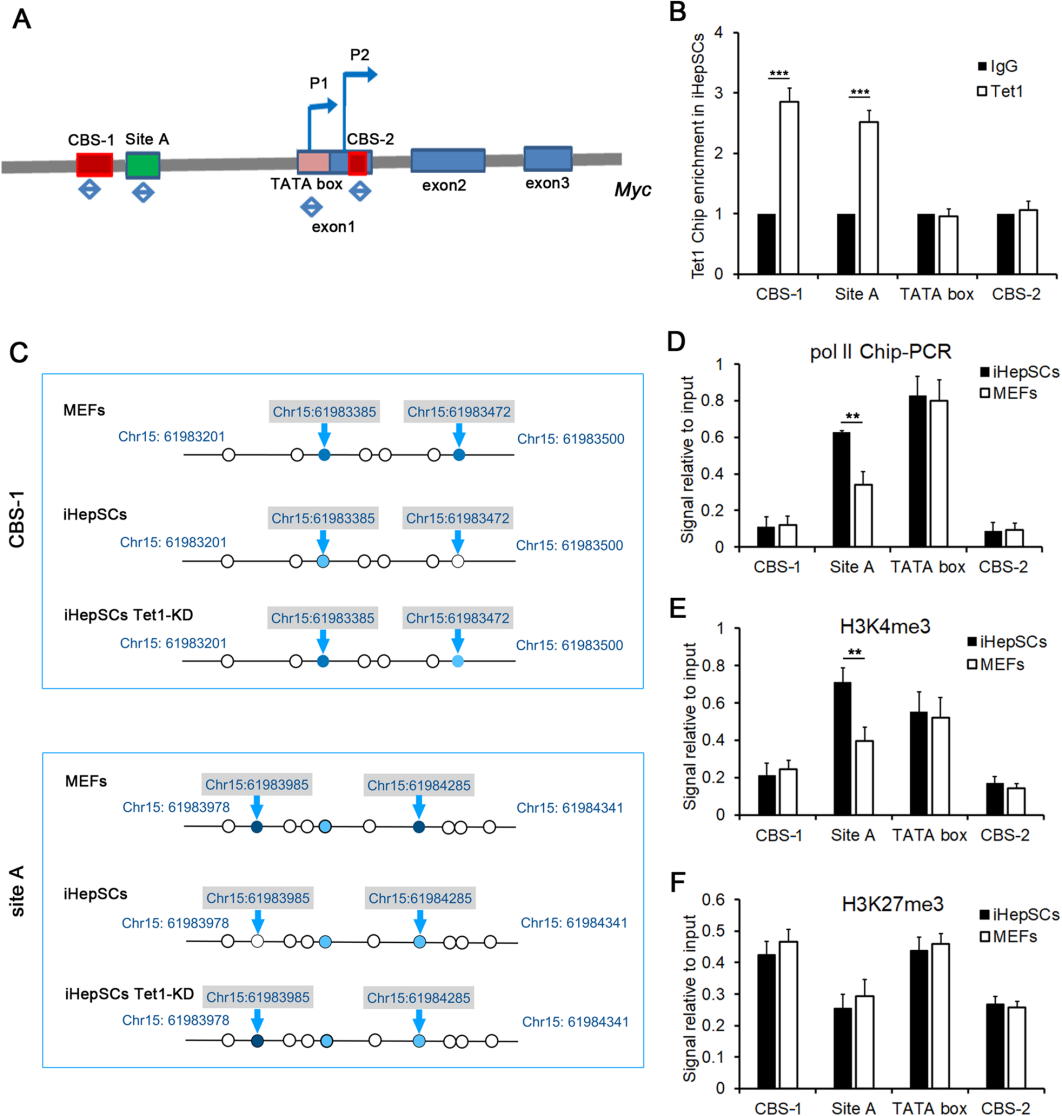
Tet1 regulates the expression of Myc by directly binding to the CBS-1 and site A regions of the Myc promoter and demethylating the CpG cytosine. **A** Schematic diagram of the position of identified *cis-*elements that participate in the regulation of Myc expression on murine chromosome 15. Red rectangles represent CBS-1 and CBS-2. Blue arrows represent two initiating sites of transcription (P1, P2). Green rectangle indicates site A. The blue double-sided arrows represent the sites of primer sets used in Tet1-ChIP PCR. **B** ChIP-PCR revealed that TET1 was enriched in the CBS-1 and site A regions rather than the TATA box and CBS-2 regions in iHepSCs (n = 6, Student’s t test, ***P < 0.001). **C** Schematic diagram of methylated CpG cytosine in the sequence of CBS-1 and site A core region in MEFs, iHepSCs and Tet1 knockdown iHepSCs detected by BSP. The levels of methylation of cytosine in both regions of iHepSCs were lower than those of MEFs and Tet1-knockdown iHepSCs. The circles represent CpG dinucleotides. The filled circle is methylated cytosine, and the empty circle represents unmethylated cytosine. Ten clones of each sample were sequenced. Circles filled with dark blue ~ 100% positive; Circles filled with light blue 50–70% positive, empty circle ~ ≤ 30% positive. **D** The results of Pol II ChIP-PCR showed that the enrichment of Polymerase II at site A in iHepSCs was higher than that in MEFs. **E** The results of ChIP-PCR showed that the enrichment of H3K4me3 at site A in iHepSCs was higher than that in MEFs (n = 6, Student’s t test, **P < 0.01). **F** The results of ChIP-PCR showed that the enrichment of H3K27me3 in iHepSCs and MEFs had no difference (n = 6, Student’s t test)

The expression of Myc is well known to be regulated by these cis elements, including CTCF binding sites (CBS-1 and CBS-2), the CSL binding site (site A), and the TATA box (Fig. 6A) [30–34]. To verify that Tet1 directly binds to the promoter region to regulate the expression of Myc, fragments of genomic DNA were precipitated with the anti-TET1 antibody. The contents of the four cis elements were quantified by qRT-PCR, and the results indicated that TET1 prefers binding to CBS-1 and the site A region rather than the TATA box and CBS-2 region in iHepSCs (Fig. 6B). Then, the methylation of cytosine in the CBS-1 and site A regions was measured by BSP, and the results showed that the level of methylation of cytosine in the CBS-1 region and site A region in Tet1 knockdown iHepSCs was higher than that in control iHepSCs, but lower than that in MEFs (Fig. 6C), which suggested that the cytosine in the CBS-1 and site A regions could be partly methylated again when the expression of Tet1 was knocked down by shRNA in iHepSCs. The results of methylation-sensitive restriction endonuclease-PCR (MSRE-PCR) also showed that only two of the CpG cytosines near the core region of CBS1 (Chr15:61,983,422–61,983,456) were methylated in MEFs but unmethylated in iHepSCs (Additional file 1: Fig. S4). Furthermore, the results of anti-pol II ChIP-PCR showed that the site A region was occupied by more polymerase II in iHepSCs than in MEFs, indicating that the promoter of Myc in iHepSCs had higher transcriptional activity (Fig. 6D). In addition, the levels of H3K4me3, which activates the transcription of genes [35], at site A in iHepSCs were also significantly higher than those in MEFs (Fig. 6E). However, there was no significant difference in H3K27me3, which suppresses transcription [35], between iHepSCs and MEFs (Fig. 6F).

### CTCF and TET1 coregulate Myc expression

**Fig. 7:**
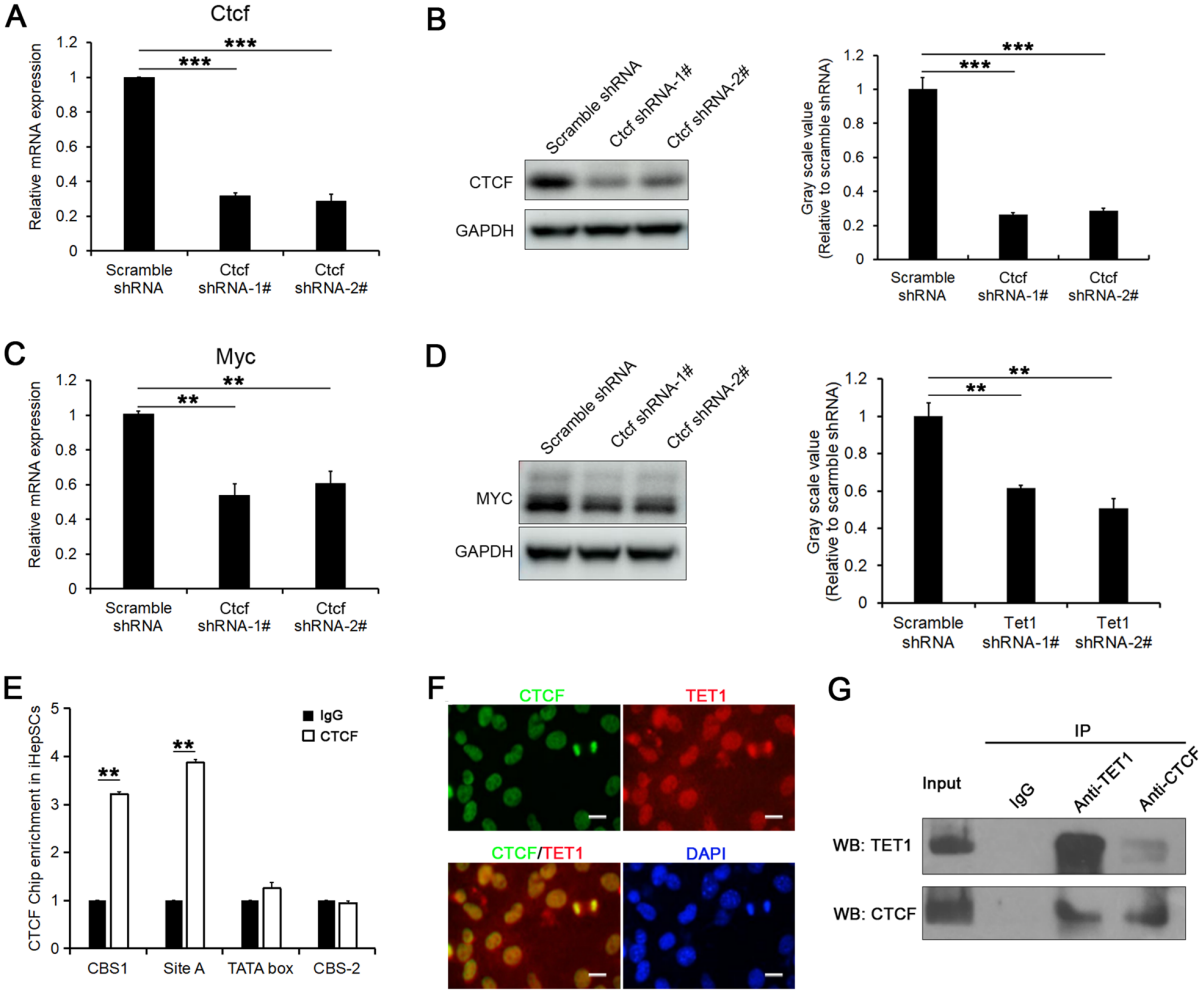
CTCF and Tet1 coregulate the expression of Myc. **A**, **B** The expression of CTCF was significantly inhibited in iHepSCs by shRNA as detected by qRT-PCR (**A**) and Western blot (**B**) (n = 3, Student’s t test, ***P < 0.001). **C**, **D** The expression of Myc was reduced in CTCF-KD iHepSCs, as detected by qRT-PCR (**C**) and Western blotting (**D**) (n = 3, Student’s t test, **P < 0.01). **E** The results of ChIP-PCR showed that CTCF was enriched in the CBS-1 and site A regions but not in the TATA box and CBS-2 regions in iHepSCs (n = 6, Student’s t test, **P < 0.01). **F** Immunocytofluorescence staining with anti-CTCF antibody and anti-TET1 antibody revealed the colocalization of CTCF protein and TET1 protein in the nucleus. **G** Co-IP assay showed that anti-TET1 antibody and anti-CTCF antibody could pull down CTCF1 and TET protein, respectively, from the nuclear extract of iHepSCs

CCCTC-binging factor (CTCF) was reported to mediate genetic or epigenetic regulatory functions, including promoter activation or repression, gene silencing, insulation, and imprinting [36–38]. In particular, CTCF was also reported to regulate the expression of Myc as a promoter activator or inhibitor in a cell type-dependent manner [30, 39]. Here, we found that the inhibition of CTCF expression in iHepSCs by shRNA also reduced the expression of Myc (Fig. 7A–D), suggesting that CTCF may promote the expression of Myc. Furthermore, we found that CTCF, like TET1, also preferentially directly combines with CBS-1 and the site A region rather than the TATA box region in iHepSCs by anti-CTCF ChIP-PCR (Fig. 7E). To further verify whether TET1 and CTCF coregulate the expression of Myc, immunofluorescence staining and co-immunoprecipitation (co-IP) assays were performed. The results of immunofluorescence staining revealed that TET1 and CTCF colocalized in the nucleus (Fig. 7F). The results of co-IP also indicated that TET1 and CTCF can form a complex to coregulate the expression of Myc (Fig. 7G).

## Discussion

Previous studies have demonstrated that active demethylation of TET proteins is indispensable to erase the original DNA methylation inheritance, establish and maintain a new DNA methylation pattern during somatic cell reprogramming and lineage reprogramming processes, and maintain a new identity of cells, including self-renewal of stem cells [40, 41]. Here, we found that compared with MEFs, the expression and activity of Tet1 in iHepSCs increased significantly, the level of cellular DNA methylation decreased and the level of hydroxymethylation increased, indicating that the new methylation patterns of iHepSCs were re-established through reprogramming from MEFs. At the same time, the current results elucidate that Tet1 plays an important role in maintaining the self-renewal of iHepSCs because downregulation of Tet1 results in obviously reduced proliferation and sphere-forming capacity in vitro.

To elucidate how Tet1 participates in the self-renewal maintenance of iHepSCs, we focused on the contribution of Myc, an essential gene in maintaining iPSC self-renewal, to stabilize the identity of iHepSCs. It is generally believed that the TET1 protein can accomplish transcription control activities either in a demethylase-dependent or demethylase-independent manner, more often as a general facilitator/recruiter of activating and repressing factors in a context-dependent manner [6]. Several studies have reported the involvement of TET1 in the self-renewal maintenance of embryonic stem cells [13, 19] and adult stem cells [20, 42]. For example, TET1 and TET2 were shown to play an important role in the proliferation of neural stem cells (NSCs) in the adult mouse brain by specifically regulating at least 16 common genes with Myc involved in DNA replication and the cell cycle [20]. However, the exact mechanism by which TET1/2 regulates Myc expression remains to be studied.

As a zinc finger transcription factor, CTCF was first identified as a transcriptional repressor of the chicken Myc gene. However, later studies revealed that CTCF is involved in Myc transcription regulation by multiple mechanisms with cell type- and cell physiology-specific mechanisms [37, 38, 43, 44]. Our current research suggests that the interaction between Tet1 and CTCF contributes to Myc expression control via the specific cis-element “site A”. Site A, a conserved CSL binding site in the promoter region marked in the Ensemble database, has been demonstrated to be the binding site of the Notch/Cdf1 complex, which directly regulates Myc expression in lymphoblastic leukemia/lymphoma and mammary tumorigenesis [45–47]. Our results provide supportive evidence that site A participates in the control of Myc expression. It is well known that the eukaryotic Pol II enzyme transcribes all protein-coding genes and noncoding regulatory RNAs (e.g., snRNA and microRNA) [34]. The formation of a preinitiation complex at the promoter, which consists of Pol II and several general transcription factors, initiates transcription. Our ChIP‒qPCR results showed that Pol II and H3K4me3 were enriched in the site A sequence, which suggested that site A may act as the transcriptional activation site in the promoter of Myc in iHepSCs, which is consistent with several recent reports [30, 32, 45].

Additionally, our results provide positive evidence of the interaction between Tet1 and CTCF through colocation assays and co-IP assays, which is in accordance with recent reports [48, 49]. Interestingly, although there is an approximately 400 bp space between site A and the core region of CBS-1, ChIP-PCR assays can detect the enrichment of TET1 on both sites in iHepSCs, and the same is true for CTCF, which may provide additional evidence for the interaction between TET1 and CTCF. In addition, the interaction may facilitate the binding of TET1 to site A and CTCF to CBS1, as well as the transcription of Myc. On the one hand, combined with a previous report about the function of CTCF as an insulator of the boundary to separate active from inactive chromatin to maintain the activation of Myc transcription [33], we postulate that the interaction between TET1 and CTCF might improve its function as an insulator by promoting or stabilizing CTCF binding with the CBS1 region with demethylation of CpG cytosine due to the oxidase activity of Tet1. On the other hand, Tet1 also promotes the demethylation of CpG cytosine at site A, which may improve the recruitment of other transcription factors at site A and the initiation of Myc transcription. These hypotheses need more research data to be supported in the future.

## Conclusion

Here, we demonstrated that the self-renewal of iHepSCs was maintained by the higher expression of Myc, which was regulated by Tet1, which directly binds to CBS-1 and site A regions of the Myc promoter and demethylates the CpG cytosine. We also confirmed that CTCF binds to the CBS-1 and site A regions of the Myc promoter and regulates Myc expression along with Tet1. This study may provide new insights into the self-renewal of stem cells, which can promote the research and application of ‘reprogrammed’ stem cells.

## Supplementary Information

**Additional file 1: Figure S1.** shRNA maintained the low expression of Tet1 and DNA methylation patterns in iHepSCs for a long time. **Figure S2.** The downregulation of Tet1 expression has no effect on the hepatic differentiation of iHepSCs. **Figure S3.** The downregulation of Tet1 expression did not affect the cholangiocytic differentiation of iHepSCs. **Figure S4.** Cytosine methylation status in CBS-1 region were analyzed by MSRE-PCR. **Table S1.** The target sequences of shRNAs. **Table S2.** Primers for qRT-PCR. **Table S3.** The exact position of cis elements of Myc gene at chromosome 15 and primer sets for ChIP. **Table S4**. Primary and secondary antibodies used in IF and Western blot assay. **Table S5.** The primers for amplifying the regions of CBS1 and Site A for BSP. **Table S6.** Primer sets for MSRE assay.

## Data Availability

All data generated or analyzed during this study are included in this published article and its Additional file.

## Abbreviations

Myc: Myelocytomatosis oncogene
Tet1: Tet methylcytosine dioxygenase 1
CTCF: CCCTC-binding factor
iHepSCs: Induced hepatic stem cells
MEFs: Mouse embryonic fibroblasts
5mC: 5-Methylcytosine
5hmC: 5-Hydroxymethylcytosine
ChIP: Chromatin immunoprecipitation
BSP: Bisulfite sequence PCR
MSRE-PCR: Methylation-sensitive restriction endonuclease-PCR
