# Supplementary material for: Induced hepatic stem cells maintain self-renewal through the high expression of Myc coregulated by TET1 and CTCF
Source: Cell Biosci. 2022 Sep 2;12:143. doi: 10.1186/s13578-022-00883-7 (PMC9440563; doi:10.1186/s13578-022-00883-7)
Supplement: Supplementary file 1 — Additional file 1: Figure S1. shRNA maintained the low expression of Tet1 and DNA methylation patterns in iHepSCs for a long time. Figure S2. The downregulation of Tet1 expression has no effect on the hepatic differentiation of iHepSCs. Figure S3. The downregulation of Tet1 expression did not affect the cholangiocytic differentiation of iHepSCs. Figure S4. Cytosine methylation status in CBS-1 region were analyzed by MSRE-PCR. Table S1. The target sequences of shRNAs. Table S2. Primers for qRT-PCR. Table S3. The exact position of cis elements of Myc gene at chromosome 15 and primer sets for ChIP. Table S4. Primary and secondary antibodies used in IF and Western blot assay. Table S5. The primers for amplifying the regions of CBS1 and Site A for BSP. Table S6. Primer sets for MSRE assay. [file 13578_2022_883_MOESM1_ESM.docx]

**Additional file**

**Induced hepatic stem cells maintain self-renewal through the high expression of Myc coregulated by TET1 and CTCF**

Chen Wang, Xinlu Yu, Sai Ding, Yang liu, Hongxia Zhang, Jingbo Fu, BingYu, Haiying Zhu


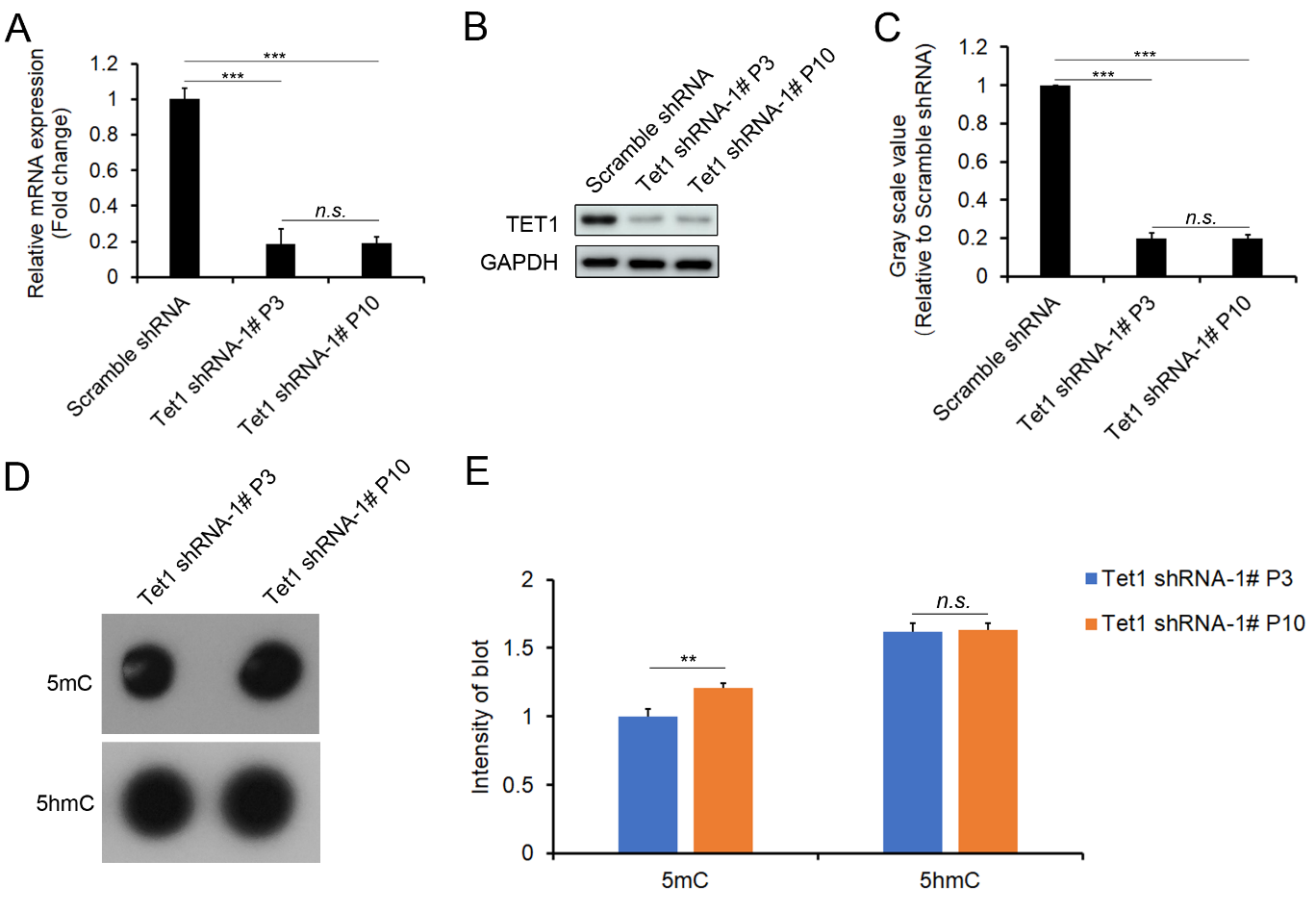


**Fig. S1** shRNA maintained the low expression of Tet1 and DNA methylation patterns in iHepSCs for a long time.

**A** The results of real-time PCR showed that there was no difference in Tet1 mRNA expression between P3 and P10 Tet1 knockdown iHepSCs. **B** The expression of Tet1 protein in P3 and P10 Tet1 knockdown iHepSCs were determined by Western Bolt. **C** The quantification of the relative intensities of blots showed that there was no difference in the expression level of Tet1 protein. **D** The levels of genomic 5mC and 5hmC were measured by dot blot. Each sample was loaded with 500 μg genomic DNA. **E** The quantification of the relative intensities of blots showed that there was no difference in the level of genomic 5hmC between P3 and P10 Tet1-KD iHepSCs. The data were shown as mean ± SEM, n = 3, **A** and **C**, Dunnett's test, **E**, Student’s t-test, ** P<0.01, ***P < 0.001, n.s.: no significant.


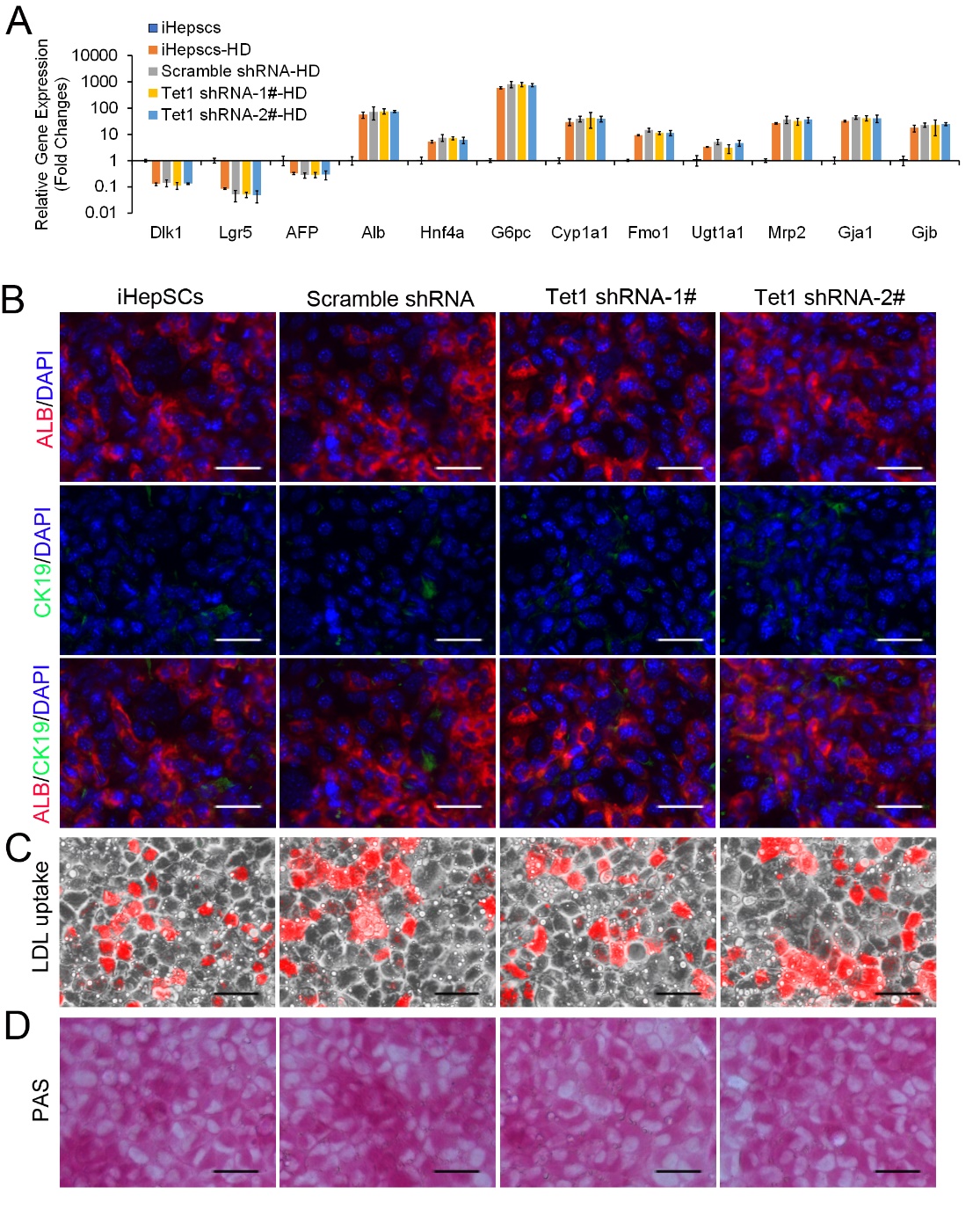


**Fig. S2** The downregulation of Tet1 expression has no effect on the hepatic differentiation of iHepSCs.

**A** After induced hepatic differentiation, the differentiated cells from each group had the similar gene expression patterns. HD, hepatic differentiation. **B** Most of the hepatic-differentiated cells in each group expressed ALB, but few of them expressed CK19. **C-D** The hepatic differentiated cells in each group had similar capacities of LDL uptake (C) and glycogen storage (D). (Scale bar: 100 μm)


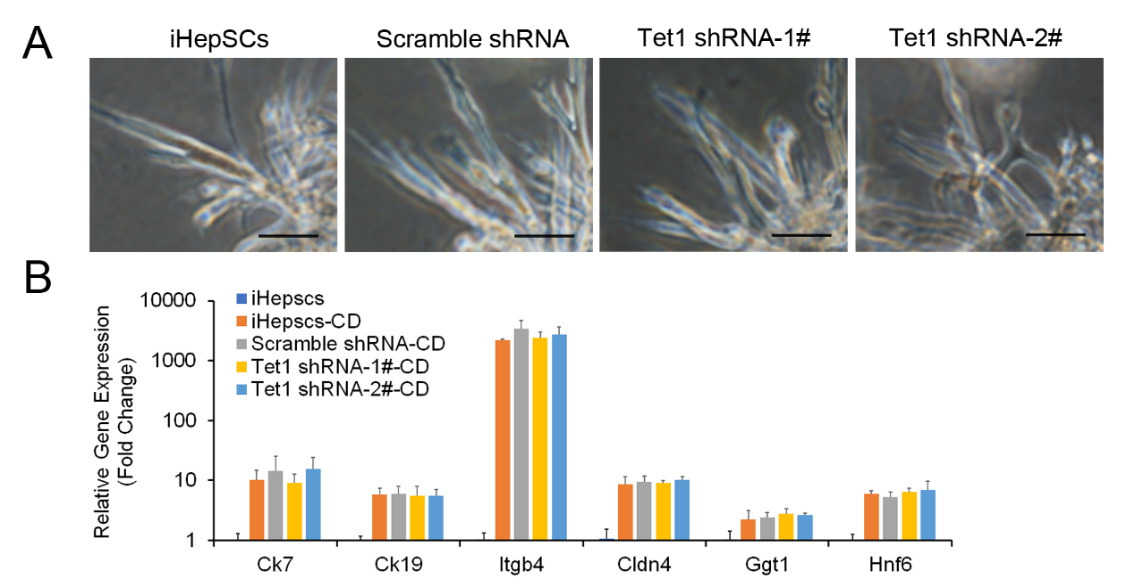


**Fig. S3** The downregulation of Tet1 expression did not affect the cholangiocytic differentiation of iHepSCs.

**A-B** After induced cholangiocytic differentiation, the differentiated cells from each group formed similar branching structures (A) and had the similar cholangiocyte-specific gene expression patterns (B). (Scale bar: 100 μm)


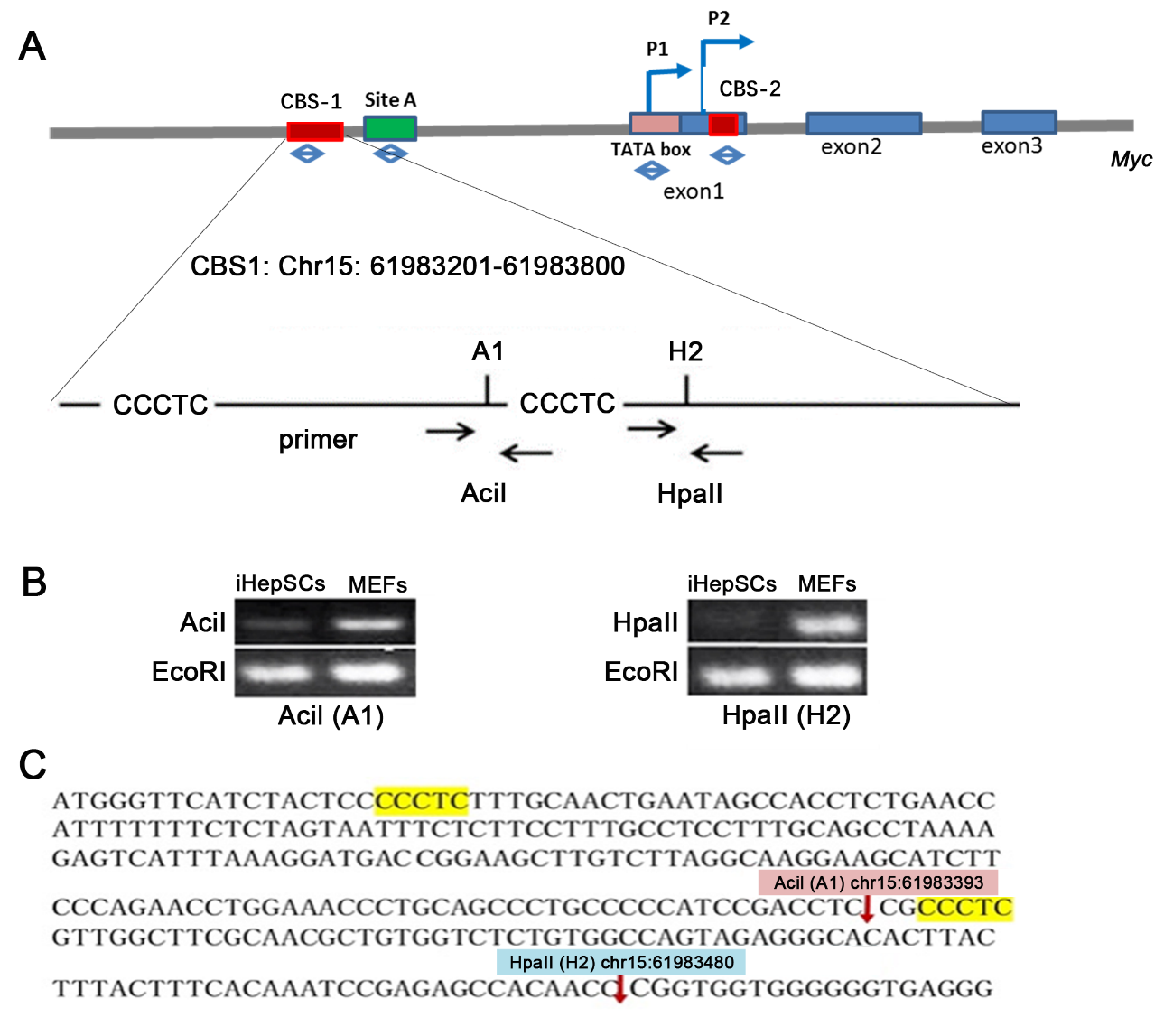


**Fig. S4** cytosine methylation status in CBS-1 region were analyzed by MSRE-PCR.

**A** Schematic diagram of methylation sensitive restriction endonuclease site at CBS-1 sequence. AciI, HpaII represent the sites of primer sets. The primers sequences listed in Table 2 amplified the fragments including AciI and HpaII digestion sites. **B** Agarose electrophoresis of MSRE-PCR products showed that the difference of cytosine methylation status in CBS-1 region between iHepSCs and MEF. Successful PCR amplification indicates the methylated cytosine; in contrast, failure of genomic DNA amplification indicates unmethylated cytosine. EcoRI digestions are used as the the negative control. The primers used in MSRE-PCR were listed in supplemental table 6. **C** A part of sequence from Chr15:61983201 to Chr15:61983500 includes AciI and HpaII site as well as two CCCTC sequences, the specific sequence of CBS.

**Table S1.** The target sequences of shRNAs.

| Targert Gene | Targert sequence |
| --- | --- |
| Tet1 shRNA-1# | CCTACGGGAAGCGACCATAAT |
| Tet1 shRNA-2# | CAACTTGCATCCACGATTAAT |
| Myc shRNA-1# | CATCCTATGTTGCGGTCGCTA |
| Myc shRNA-2# | CGTGACCAGATCCCTGAATTG |
| Ctcf shRNA-1# | GCGAAGAATGACCACAAAT |
| Ctcf shRNA-2# | TGGACGATACCCAGATCATAA |

**Table S2.** Primers for qRT-PCR

| Gene name | Forward primer sequence | Reverse primer sequence |
| --- | --- | --- |
| Tet1 | TCATTCCAGACCGCAAGACC | TGACACCAGAGAAAGGACGC |
| Tet2 | AGAGCCTCAAGCAACCAAAA | ACATCCCTGAGAGCTCTTGC |
| Tet3 | CCGGATTGAGAAGGTCATCTAC | AAGATAACAATCACGGCGTTC |
| Myc | TCTGCTCTCCATCCTATGTT | CCAAGTAACTCGGTCATCAT |
| Ctcf | AGCGCTATCATGATCCCAAC | CGGCTCAGCATTTTCTTCAC |
| Dlk1 | CCCAGGTGAGCTTCGAGTG | GGAGAGGGGTACTCTTGTTGAG |
| Lgr5 | CAGCCTCAAAGTGCTTATGC | GCGTAGTCTGCTATGTGGTGTA |
| Afp | CTTCCCTCATCCTCCTGCTAC | ACAAACTGGGTAAAGGTGATGG |
| Alb | CCACTGTTGAAGAAAGCCCA | CAGATAGTCTTCCACACAAGGCA |
| Hnf4a | ACACGTCCCCATCTGAAGGTG | CTTCCTTCTTCATGCCAGCCC |
| G6pc | CGACTCGCTATCTCCAAGTGA | GTTGAACCAGTCTCCGACCA |
| Cyp1a1 | ATGTCATCTGTGCCATATGCTT | TGTGGCCCTTCTCAAATGTCC |
| Fmo1 | GTATAACAAAACGCCCGGAT | CGCTTGTCCTTAAATATGTCT |
| Ugt1a1 | TACACCGGAACTAGACCATCG | TTGGCATTGTCCATCTGATCG |
| Mrp2 | TCGTAACAGAAGTGCCCTGG | AACACCTGCTTGGCAAGGTA |
| Gja1 | TCCTTCAGATCATATTCGTGTC | GCAGCCATTGAAGTAAGCAT |
| Gjb1 | CGTGAATCGGCACTCTACAGC | TGCACCTTGTGTCTCTTTACCTC |
| Ck7 | AATTCGCCTCCTTCATCGACA | CCAACTCCACCTTGTTCGTGT |
| Ck19 | GTCCTACAGATTGACAATGC | CACGCTCTGGATCTGTGACAG |
| Itgb4 | GCAGACGAAGTTCCGACAG | GGCCACCTTCAGTTCATGGA |
| Cldn4 | GTCCTGGGAATCTCCTTGGC | TCTGTGCCGTGACGATGTTG |
| Ggt1 | TTTGTCATCATCGGCCTCTGT | CCCGTCCAATCTCTGAGCAG |
| Hnf6 | CAGCATCCCACAGGCCATCTT | TCTCCCGACCCGACTTGAG |
| Gapdh | GGTGAAGGTCGGTGTGAACG | CTCGCTCCTGGAAGATGGTG |

**Table S3.** The exact position of cis elements of Myc gene at chromosome 15 and primer sets for ChIP.

| *cis*-element | position at chr15 | primer sets for ChIP |
| --- | --- | --- |
| CBS-1 | 61983200-62983800 | F: GCAGCAAAACGCAGACTAGG  R: CTTCATCGAGCTCATTGCACA |
| Site A | 61983978-61984341 | F: GGAAACTGGGAAATTAATGTA  R: TTCCCAGAAAGGGGGAGGAGTGA |
| Primer 1 (P1) | 61985203-61985378 | F: ACTAGCGCGCGAGCAAGAGAAAA  R: GGGATTAGCCAGAGAATCTCTCT |
| Primer 2 (P2)  （CBS-2） | 61985547-61985599 | F: CCTTTATATTCCGGGGGTCT  R: AGAGACAG AGGGAGTGAGCG |

The corresponding exact site listed in Supplemental table 3 are located according to UCSC database.

**Table S4**. Primary and secondary antibodies used in IF and Western blot assay.

| **Antigen** | **Cat#** | **Company** |
| --- | --- | --- |
| 5mC | A-1014 | Epigentek |
| 5hmC | A-1018 | Epigentek |
| CTCF | #3418 | Cell Signaling |
| Myc | #5605 | Cell Signaling |
| GADPH | #2118 | Cell Signaling |
| Alpha Tubulin | 66031-1-Ig | Proteintech |
| Tet1 | sc-163446 | Santa Cruz |
| FITC AffiniPure Goat Anti-Mouse IgG (H+L), | 115-095-003 | Jackson ImmunoResearch, |
| Cy™5 AffiniPure Goat Anti-Mouse IgG (H+L) | 115-175-146 | Jackson ImmunoResearch, |
| FITC AffiniPure Donkey Anti-Rabbit IgG (H+L) | 711-095-152 | Jackson ImmunoResearch, |
| Cy™5 AffiniPure Donkey Anti-Goat IgG (H+L) | 705-175-147 | Jackson ImmunoResearch, |
| HRP-Goat anti mouse IgG | sc-2005 | Santa Cruz |
| HRP-Goat anti rabbit IgG | sc-2004 | Santa Cruz |

**Table S5.** The primers for amplifying the regions of CBS1 and Site A for BSP.

| *cis*-element | position at chr15 | primer |
| --- | --- | --- |
| CBS-1 | 61983200-62983800 | F: GTAGTTTAAAAGAGTTATTTAAAGGATG  R: AAAACCACCAATCCCTAATCTAC |
| Site A | 61983978-61984341 | F: TTTATGTTTTTGGTTGGTTAATAAG  R: CCTATAAAACCAATTTACAACAAAC |

**Table S6.** Primer sets for MSRE assay

| restriction endonuclease | Primer sets |
| --- | --- |
| AciI | F: CTCTTCCTTTGCCTCCTTTG  R: GTTGTGGCTCTCGGATTTGT |
| HpaII | F: TGTGGTCTCTGTGGCCAGTA  R: GCCTCTAGGATTTTGCCTCA |
